# Identification and characterization of whole blood gene expression and splicing quantitative trait loci during early to mid-lactation of dairy cattle

**DOI:** 10.1186/s12864-024-10346-7

**Published:** 2024-05-06

**Authors:** Yongjie Tang, Jinning Zhang, Wenlong Li, Xueqin Liu, Siqian Chen, Siyuan Mi, Jinyan Yang, Jinyan Teng, Lingzhao Fang, Ying Yu

**Affiliations:** 1 Key Laboratory of Animal Genetics, Breeding and Reproduction, Ministry of Agriculture & National Engineering Laboratory for Animal Breeding, College of Animal Science and Technology, China Agricultural University, Beijing, 100193 China; 2 Center for Quantitative Genetics and Genomics, Aarhus University, Aarhus, 8000 Denmark; 3 State Key Laboratory of Swine and Poultry Breeding Industry, National Engineering Research Center for Breeding Swine Industry, Guangdong Provincial Key Lab of Agro-Animal Genomics and Molecular Breeding, College of Animal Science, South China Agricultural University, Guangzhou, 510642 China

**Keywords:** Holstein cows, Early to mid-lactation period, eQTL, sQTL, Colocalization, TWAS

## Abstract

**Background:**

Characterization of regulatory variants (e.g., gene expression quantitative trait loci, eQTL; gene splicing QTL, sQTL) is crucial for biologically interpreting molecular mechanisms underlying loci associated with complex traits. However, regulatory variants in dairy cattle, particularly in specific biological contexts (e.g., distinct lactation stages), remain largely unknown. In this study, we explored regulatory variants in whole blood samples collected during early to mid-lactation (22–150 days after calving) of 101 Holstein cows and analyzed them to decipher the regulatory mechanisms underlying complex traits in dairy cattle.

**Results:**

We identified 14,303 genes and 227,705 intron clusters expressed in the white blood cells of 101 cattle. The average heritability of gene expression and intron excision ratio explained by *cis*-SNPs is 0.28 ± 0.13 and 0.25 ± 0.13, respectively. We identified 23,485 SNP-gene expression pairs and 18,166 SNP-intron cluster pairs in dairy cattle during early to mid-lactation. Compared with the 2,380,457 *cis*-eQTLs reported to be present in blood in the Cattle Genotype-Tissue Expression atlas (CattleGTEx), only 6,114 *cis*-eQTLs (*P* < 0.05) were detected in the present study. By conducting colocalization analysis between *cis*-e/sQTL and the results of genome-wide association studies (GWAS) from four traits, we identified a *cis*-e/sQTL (rs109421300) of the *DGAT1* gene that might be a key marker in early to mid-lactation for milk yield, fat yield, protein yield, and somatic cell score (PP4 > 0.6). Finally, transcriptome-wide association studies (TWAS) revealed certain genes (e.g., *FAM83H* and *TBC1D17*) whose expression in white blood cells was significantly (*P* < 0.05) associated with complex traits.

**Conclusions:**

This study investigated the genetic regulation of gene expression and alternative splicing in dairy cows during early to mid-lactation and provided new insights into the regulatory mechanisms underlying complex traits of economic importance.

**Supplementary Information:**

The online version contains supplementary material available at 10.1186/s12864-024-10346-7.

## Background

Thousands of genetic variants were discovered to be associated with complex traits in cattle through genome-wide association studies (GWAS) [1]. However, most of these variants are not coding variants; therefore, understanding the molecular mechanisms behind these GWAS loci is challenging [2]. Previous studies have shown that genetic variation can affect gene expression and splicing, often termed gene expression quantitative trait loci (eQTL) and splicing QTL (sQTL). This has led to a focus on two important classes of regulatory variants [3]. Identification of eQTL and sQTL is important for understanding the relationship between regulatory variants and complex traits and has acquired progress in bovine studies. FarmGTEx consortium built a Cattle Genotype-Tissue Expression atlas (CattleGTEx) which included numerous eQTLs and sQTLs associated with complex traits in different tissues [4]. Additionally, the eQTL and sQTL explained a large proportion of the heritability of complex traits in cattle [5]. However, little is known about how allelic variation affects regulatory interactions during early to mid-lactation in dairy cattle.

Critically, the genetic effect of regulatory variants is highly context-dependent [6, 7], consistent with transcriptional surveys of dairy cow lactation that show prominent temporal changes in gene expression [8, 9]. Early to mid-lactation is the key period in the dynamic lactation process of dairy cows, and determines milk production and health performance [10, 11], thus highlighting the need to identify regulatory variation within this critical time point. As whole blood is the most easily obtained specimen, it is widely used to comprehensively study the mechanisms of complex traits. Whole blood can reflect the physiological conditions of cows as it is responsible for transporting various substances used in milk production [12]. Some studies have shown that individuals with different milk production performances have different gene expression levels, and potential molecular biomarkers in the blood transcriptome related to milk performance traits have been identified [12, 13]. In addition, blood leukocytes are widely used as immune cells in transcriptional surveys of health traits, such as mastitis [14] and ketosis [15]. Notably, the identification of regulatory QTLs using whole blood has also revealed a correlation between the genetic effects of blood and other tissues [2, 16]. The genetic effect of some regulatory QTLs is shared among different tissues [3, 17–19].

Therefore, the aim of this study was to identify eQTL and sQTL in the early to mid-lactation period of dairy cows using whole blood and to explore their association with complex traits. We hope that this study will provide insight into changes observed in the effects of genes on complex traits during lactation from the perspective of the regulatory roles that some variants play in gene expression and splicing.

## Results

### Identification of factors affecting gene expression and intron excision ratio

In this study, whole blood leukocytes from 104 Holstein cattle in early to mid-lactation (22–150 days after calving) were genotyped and RNA-Seq was performed. After quality control and normalization of gene expression and genotypes, 95,799 SNPs, 14,303 genes and 227,705 intron clusters from 101 individuals were obtained for eQTL and sQTL identification and characterization [Additional file 2, Figure S1].

As an intermediate molecular phenotype, transcripts are affected by confounding factors such as batch effects and biological and technical factors [4, 6]. The results suggested that the week of lactation, parity and RIN showed stronger correlations with gene expression principal components (EPCs) and intron excision ratio principal components (SPCs) than blood cell counts [Additional file 2, Figure S2]. In addition to the above-mentioned known factors affecting transcription level changes, the PEER software was used to identify unknown confounding factors (PEER factors) [4, 20]. The factor weight variances near zero when the number of hidden PEER factors inferred from gene expression and intron cluster expression reached 10 and 8, respectively [Additional file 2, Figure S3]. Therefore, the top ten PEER factors were removed from the eQTL discovery, and the top eight PEER factors were removed from sQTL discovery.

### Heritability of gene expression and intron excision ratios

Gene expression and intron excision ratios with heritability (*h*^2^) > 0 and *P* < 0.05, respectively, were considered heritable. There are 4,604 genes whose expression with heritability that can be explained by *cis*-SNPs (*h*^2^ > 0, *P* < 0.05), and the heritability was 0.28 ± 0.13 (mean ± standard deviations). Meanwhile, there are 21,983 intron excision ratios with heritability that can be explained by *cis*-SNPs (*h*^2^ > 0, *P* < 0.05), and the heritability was 0.25 ± 0.13 [Additional file 2, Figure S4].

### Identification of *cis*-eQTLs and *cis*-sQTLs during early to mid-lactation of dairy cows

FastQTL was used to identify *cis*-eQTL and *cis*-sQTL, adjusting for known (week of lactation, parity, RIN, and genotype PCs) and inferred covariates (PEER factors). This resulted in 23,485 SNP-gene expression pairs (*FDR* < 0.05) [Additional file 1, Table S1] and 18,166 SNP-intron cluster pairs (*FDR* < 0.05) [Additional file 1, Table S2]. Among them, 3,419 genes had significant eQTL and 3,127 genes had significant sQTL, hereafter referred to as eGenes and sGenes respectively.

### Differences in genomic features between eQTLs and sQTLs

**Fig. 1:**
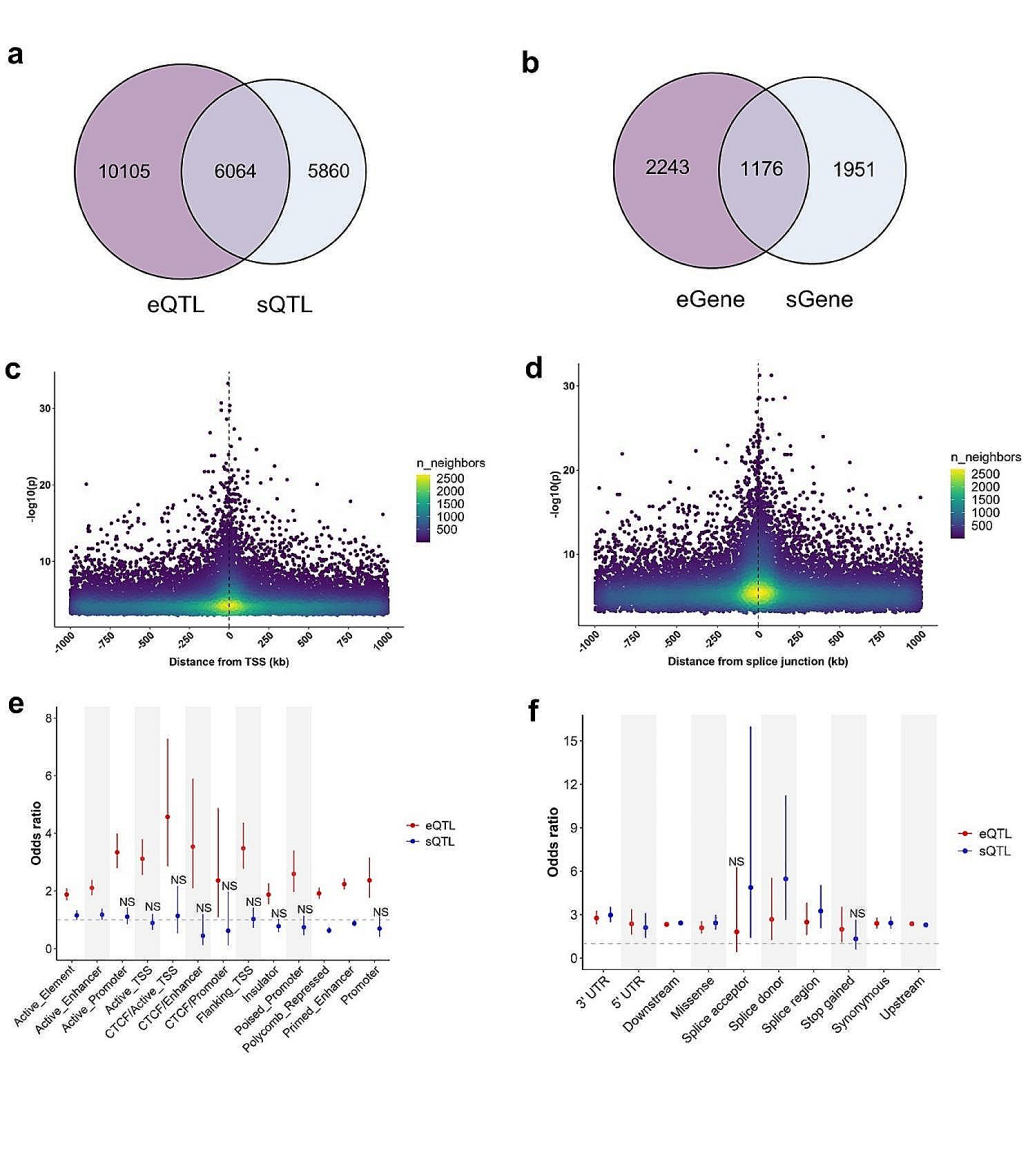
Comparison of *cis*-eQTLs and *cis*-sQTLs characterization. (**a**) Overlap of eQTLs and sQTLs. (**b**) Overlap of eGenes and sGenes. (**c**) Distance of eQTL in early-mid lactation dairy cows relative to TSS of eGene. Each point represents an eVariant-eGene pair (*FDR* < 0.05). (**d**) Distance of sQTL relative to splice junction of targeted intron cluster. Each point represents an sVariant-sGene pair. (*FDR* < 0.05). (**e**) Enrichment (Fisher’s exact test) of eQTLs and sQTLs with 13 chromatin states in the spleen, respectively. The point and error bars indicate the odds ratio and 95% CI. (**f)** Enrichment (Fisher’s exact test) of eQTLs and sQTLs with Ensembl VEP-predicted SNP effects, respectively. The point and error bars indicate the odds ratio and 95% CI

The overlap of eQTLs with sQTLs was further analyzed, as well as for eGenes and sGenes. The results suggested that nearly half of the sQTLs (approximately 49.1%) were not eQTL and approximately 62.5% of the eQTLs were independent (Fig. 1a). In addition, only 34.4% of the eGenes were also sGenes and 37.6% of the sGenes were also eGenes (Fig. 1b). Even when the eGene was also an sGene, the lead QTL SNPs distances were mostly between 10 kb and 1 Mb [Additional file 2, Figure S5a] and in low linkage disequilibrium (LD) r^2^ [Additional file 2, Figure S5b].

Next, the results suggest that most of the eQTLs were located near the TSS and were more significant than others (Fig. 1c), whereas the sQTLs tended to be located near the splice junction (Fig. 1d). Therefore, the results suggest that genetic variation near the TSS (promoter, etc.) has a large effect on cognate gene expression, whereas genetic variation near the splice junction is more likely to affect gene alternative splicing. Furthermore, enrichment analysis (Fisher’s exact test) was conducted comparing the 13 chromatin states of cow spleen with eQTLs and sQTLs. It was found that eQTLs tend to be more enriched in transcriptional regulatory elements such as active enhancer and TSS compared to sQTLs (Fig. 1e). Additionally, enrichment analysis (Fisher’s exact test) was separately performed on eQTLs and sQTLs using Ensembl VEP-predicted SNP effects, revealing that sQTLs tend to be more enriched in splicing-related regions compared to eQTLs (Fig. 1f).

### Specificity of eQTL during early to mid-lactation of dairy cows

**Fig. 2:**
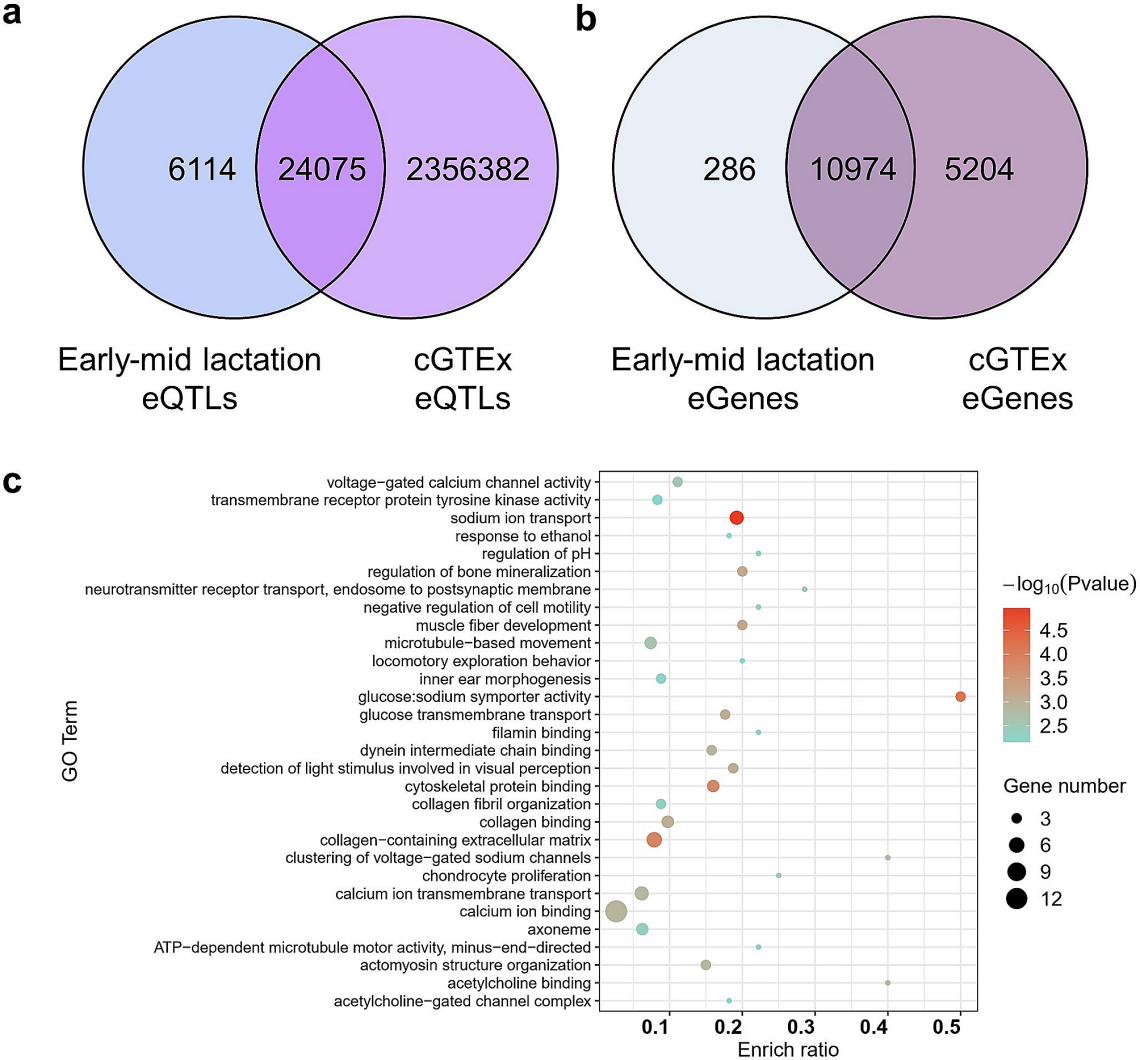
Comparison of eQTLs and eGenes in this study and GTEx. (**a**) Comparison of *cis*-eQTLs in early-mid lactation and cGTEx (*P* < 0.05). (**b**) Comparison of eGenes in early-mid lactation and cGTEx. (**c**) GO enrichment analysis of eGenes specific to early-mid lactation in this study

To identify eQTLs and eGenes specific for early to mid-lactation in Holstein cows, the *cis*-eQTLs and eGenes were compared with the population in cGTEx. The results suggested that there were 24,075 common eQTLs and 6,114 specific eQTLs in the early to mid-lactation (*P* < 0.05; Fig. 2a). In terms of eGenes, the shared number of eGenes is 10,974, and the number of specific eGenes in early-mid lactation is 286 (*P* < 0.05; Fig. 2b). These 286 genes were enriched in metabolic pathways related to sodium, calcium and glucose transport (*P* < 0.01; Fig. 2c).

### Gene co-expression network of eGenes during early to mid-lactation cows

**Fig. 3:**
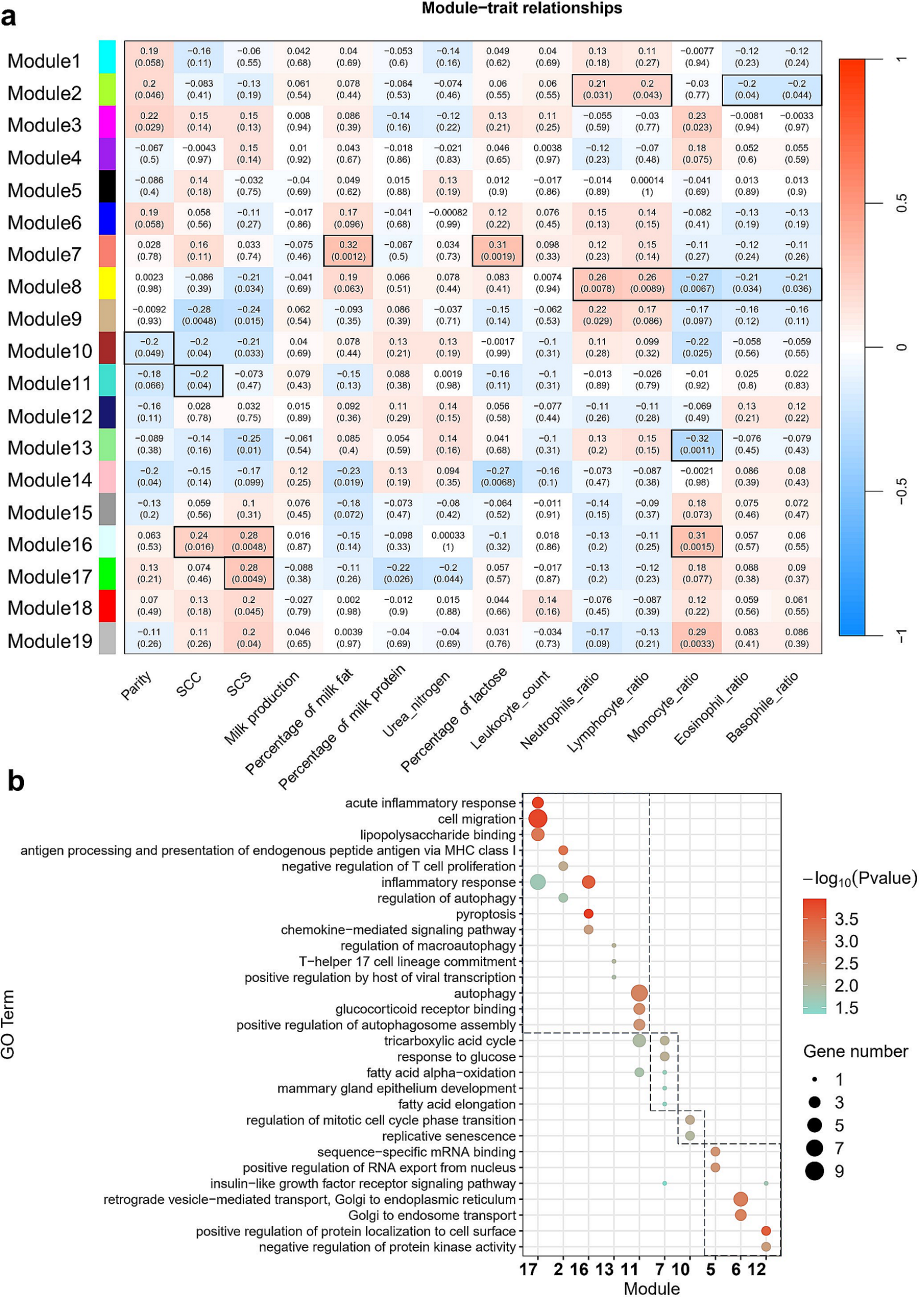
Characterizing the function of gene expression modules. (**a**) Association between eGene co-expression module and individual phenotype of trait. (**b**) GO analysis of phenotype of trait significant association module genes. GO terms of modules related to function of immune (module 17,2,16,13 and 11), metabolic (module 7), parity (module 10) and gene regulation (module 5,6 and 10). SCC: somatic cell counts. SCS: somatic cell score

To explore the biological functions of all eGenes during early to mid-lactation cows in this study, 3,419 eGenes (Fig. 1e, *FDR* < 0.05) were used to construct a gene co-expression network, and 18 co-expression modules of eGenes in early to mid-lactation blood were identified (Fig. 3a). Next, 18 modules were tested for association with phenotypic traits (parity, somatic cell count, somatic cell score, milk production, percentage of milk fat, percentage of milk protein, urea nitrogen, percentage of lactose, leukocyte count, and neutrophil, lymphocyte, monocyte, eosinophil, and basophil ratios). The results suggested that different modules were significantly associated with known phenotypes (*P* < 0.05; Fig. 3a).

Genes in the identified modules were enriched in GO terms corresponding to the biological features of the phenotype (Fig. 3b). Five modules (modules 2, 11, 13, 16, and 17) that were significantly correlated with SCC, SCS, and blood parameters were enriched for immune-relevant GO terms, such as response to acute inflammatory response, lipopolysaccharide binding, and regulation of autophagy (Fig. 3b). One module (module 7) was significantly correlated with the milk fat rate and lactose rate, which is enriched for metabolism relevant GO terms, such as tricarboxylic acid cycle and response to glucose (Fig. 3b). One module (module 10) was significantly correlated with parity and was enriched for longevity and development-relevant GO terms, such as replicative senescence (Fig. 3b). In addition, modules 5, 6, and 12 were not found to be significantly associated with known phenotypes, but these modules play a role in regulating functions such as sequence-specific mRNA (Fig. 3b).

### Colocalization analysis of eQTL and sQTL with GWAS locus

**Fig. 4:**
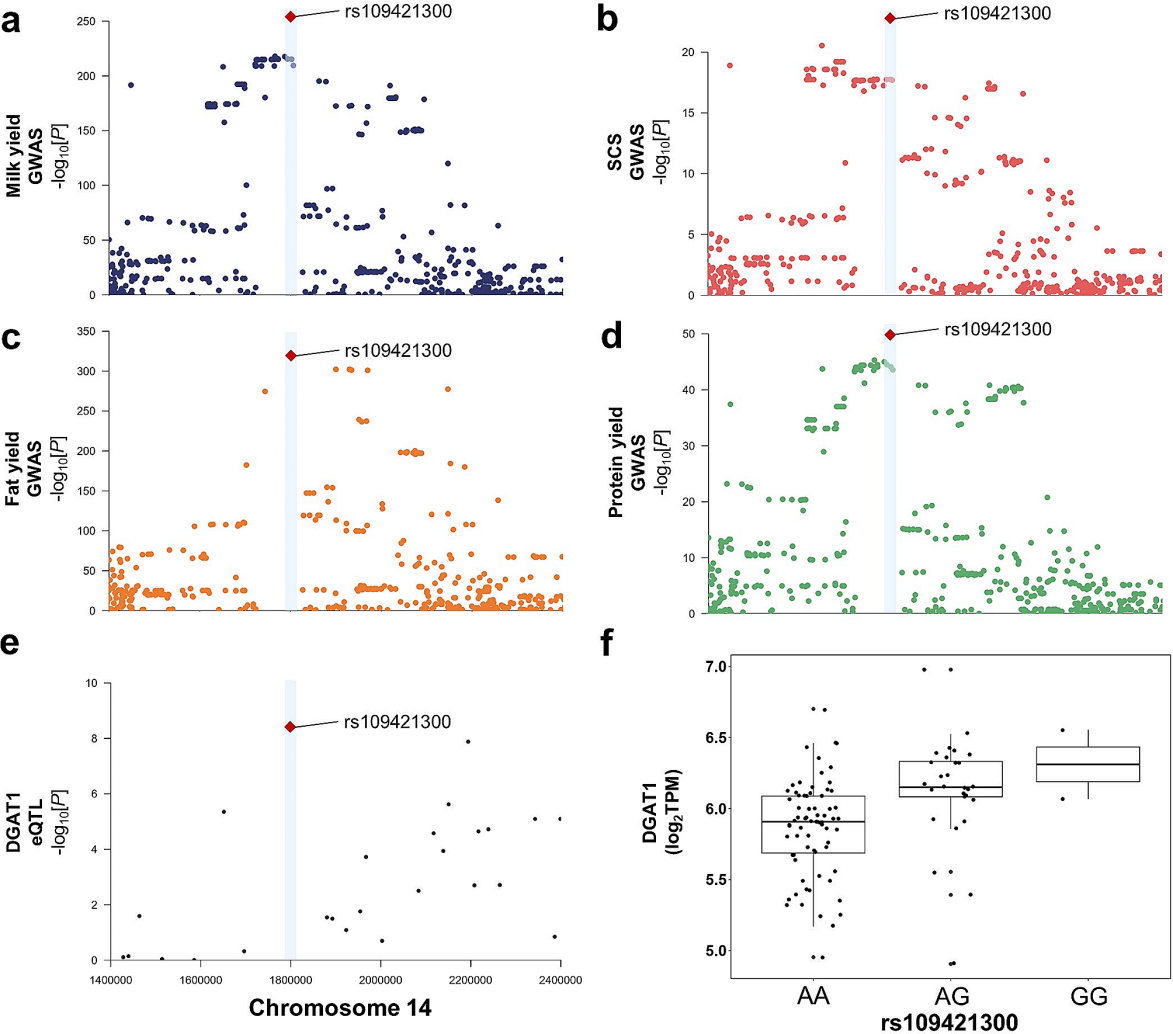
GWAS signals of *DGAT1* gene co-localized with eQTL and sQTL in four traits. (**a-d**) are GWAS Manhattan plots of milk yield, somatic cell score, milk fat and milk protein respectively. (**e**) Manhattan plot of eQTL. (**f**) The allele of eQTL rs109421300 corresponds to the expression level of *DGAT1* gene. The selected genome range of Manhattan plot is consistent: Chr14:1.4-2.4 Mb. The reference genome is UMD3.1

The eQTL and sQTL identified in this study were co-localized with three production traits (milk yield, milk protein yield, and milk fat yield) and one health trait (somatic cell score) GWAS loci in 27,214 dairy cows [21]. The results suggest that SNP rs109421300, which was significantly associated with milk yield, milk protein yield, milk fat yield, and somatic cell score, was also an eQTL and sQTL of *DGAT1* (Fig. 4). The *DGAT1* gene is known to be an important gene for production traits in dairy cows and plays a key role in regulating milk fat production. Previous study has reported the known K232A coding mutation (rs109234250 and rs109326954) in *DGAT1* [22]. Through linkage disequilibrium analysis, it was found that rs109421300 is highly linked with the K232A coding mutation (Figure S6a). Meanwhile, conditional analysis of *DGAT1* gene expression with rs109421300 as a covariate still revealed an independent eQTL (Figure S6b). Therefore, the biological effects of SNPs related to the *DGAT1* gene need further validation. SNP rs109421300 may be a key marker related to *DGAT1* gene expression, alternative splicing, and individual phenotypic traits.

### TWAS

**Fig. 5:**
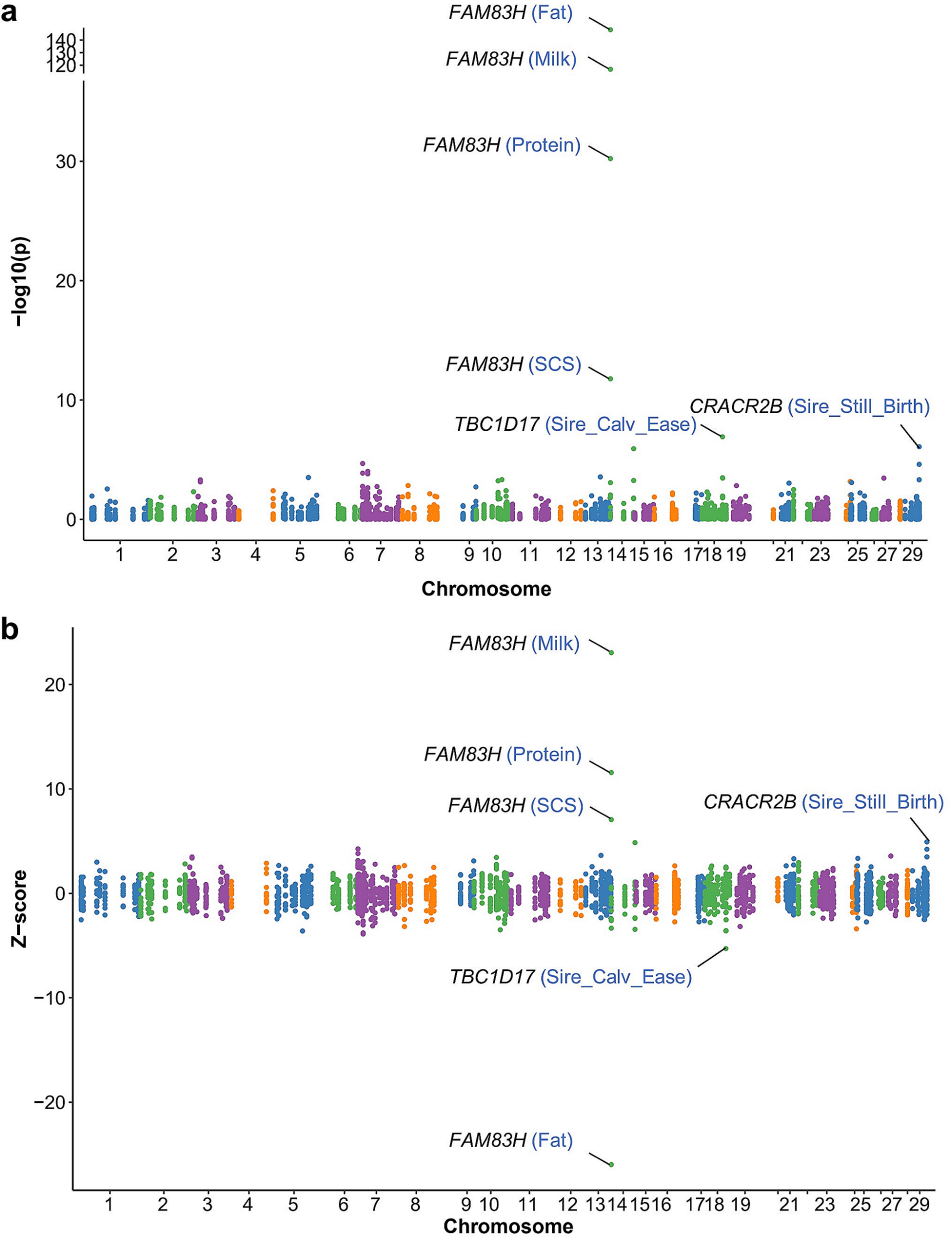
TWAS. (**a**) Gene-level Manhattan plot showing *P*-value results from TWAS. (**b**) *Z*-scores showing the direction of genetic effect for the genotype-inferred expression of transcripts. Sire_Calv_Ease: Sire calving ease; Sire_Still_Birth: Sire stillbirth. The reference genome is ARS.UCD.1.2

To explore the association between gene expression and complex traits, we integrated the SNP genotyping, gene expression, and GWAS summary data. These results suggest that *FAM83H* gene expression was significantly associated with milk fat yield, milk yield, milk protein yield, and SCS traits (*P* < 0.05; Fig. 5a) [Additional file 1, Table S3]. The effect of *FAM83H* gene expression level on milk fat yield was the opposite of that on milk yield, milk protein yield, and somatic cell count (Fig. 5b). Genes *TBC1D17* associated with sire calving ease, and *CRACR2B* associated with sire stillbirth, were also identified (*P* < 0.05).

## Discussion

Here, we reveal a part of the genetic control pattern of gene expression and splicing in dairy cows during early to mid-lactation, and highlight the impact of regulatory variation on complex traits.

Transcripts, as molecular phenotypes, are susceptible to confounding factors, such as biological and technical factors [23]. Therefore, this study systematically evaluated the influence of confounding factors to ensure the robustness and reproducibility of the eQTLs and sQTLs. Previous studies have showed that the lactation stage [9] and parity [24] of dairy cattle, as well as RIN [25] and cell type composition [26], affect transcript expression. Similarly, this study also found that lactation stage, parity, and RIN value need to be considered as covariates in the association analysis of blood.

In this study, most of the eQTLs and sQTLs identified during early to mid-lactation of dairy cows, as well as the eGenes and sGenes, were independent. This indicates that there are similarities and differences in the regulation of gene expression and alternative splicing mechanisms by molecular QTL. A recent study showed that the overlap ratio of detected eGenes and sGenes positively correlated with the number of samples [27]. However, even if a gene is both an eGene and sGene, most of the corresponding eQTLs and sQTLs are far away from each other and have a low degree of linkage. It is worth noting that limited to the short read length of next-generation sequencing, we do not have effective analysis methods to completely identify each alternatively spliced isoform and to understand the regulatory mechanism of sQTL.

Early to mid-lactation is an important period for dairy cattle production. By comparing eQTLs and eGenes in early-mid lactation with cGTEx, some eQTLs and eGenes with specific effects during early to mid lactation were found. GO terms enriched by these specific eGenes are interesting, for example, glucose can affect milk protein synthesis [28], and calcium is essential for milk synthesis [29]. However, it should be noted that the number of SNPs, sample size, composition of breeds, and other information used in this study and in cGTEx for eQTL identification differ. Therefore, this study focuses on the specific eQTLs and eGenes in the early and mid-lactation periods of Holstein cows. In addition, WGCNA analysis of eGenes in early to mid-lactation showed that the eGene expression module was enriched in the biological function module, corresponding to the individual phenotype. Although blood may not be the main tissue for functions other than immunity, it potentially contains genetic regulatory information on various tissues and organs in an individual [30, 31]. Because the effects of eQTLs detected in blood may be shared across multiple tissues, the contribution of these eQTLs to the phenotype of complex traits is completed through gene expression control in multiple tissues [3, 4, 32].

Colocalization is an effective method for integrating molecular QTLs with GWAS signals of complex traits to identify possible causal mutations. In this study, rs109421300 was a key marker obtained by co localization of eQTL and sQTL of *DGAT1* with GWAS signals. Meanwhile, rs109421300 is also a *cis*-eQTL for *DGAT1* in the blood, liver, macrophages, mammary glands, monocytes, pituitary glands, and uterus of the cGTEx atlas but not *cis*-sQTL [4]. This indicated that the eQTL effect of rs109421300 may not be limited to early-mid lactation, but that the sQTL effect may be specific to early-mid lactation. *DGAT1* is important for lactation in dairy cows [33, 34]. Knockdown of *DGAT1* expression in mammary epithelial cells significantly reduced intracellular triglyceride content [35]. Regarding for the SNP effect, among the A and G alleles of rs109421300, the G allele resulted in extreme antagonistic pleiotropy between positive milk fat yield, negative milk yield, and milk protein yield [36]. However, the effect of rs109421300 is based on association analysis, which could potentially be influenced by linkage disequilibrium. As a result, further validation through FLGA [37] and dual-luciferase reporter assay systems is needed to confirm its biological effects.

It is worth noting that rs109421300 is located 1,149 bp upstream of the reported K232A causal mutation of *DGAT1*. K232A affects the activity of DGAT1 enzyme and alternative splicing of *DGAT1* [22, 38], while rs109421300 is located in a non-coding region, which is mainly observed to be associated with gene expression and alternative splicing. We hypothesize that non-coding variants and K232A coding mutation may be different biological factors affecting *DGAT1* gene. However, due to the limitation of the SNP beadchip lacking information on the K232A coding mutation, further study is needed to investigate the differential impact of both rs109421300 and K232A on *DGAT1* gene expression and alternative splicing. Given the high linkage of SNPs around the *DGAT1* gene and the complexity of its regulatory network, as well as the importance of *DGAT1* in dairy cow production, future studies should systematically analyze the regulation of genomic variations on target genes through epigenetic regulatory elements, three-dimensional genomics, luciferase reporter gene assays, and gene editing (e.g. CRISPR-Cas9) [39].

The TWAS is an important method for inferring the causal relationship between gene expression and the phenotypes of complex traits [40]. We found that *FAM83H* was significantly associated with milk fat yield, milk yield, milk protein yield, and SCS. *FAM83H* was also found to be regulated by eQTL in other blood samples from dairy cows [4, 41]. Interestingly, the direction of the effect of *FAM83H* expression on milk fat yield, milk protein yield, milk yield, and SCS was similar to that of *DGAT1*, and their effects on the phenotypes of complex traits may be genetically linked. In addition, dairy cattle are involved in pregnancy events during early to mid-lactation; therefore, the development of early embryos and reproductive organs may be related to sire calving ease and sire stillbirth. *TBC1D17* associated with sire calving ease is a member of the TBC1 domain family, and studies have shown that *TBC1D2* can be used as a diagnostic tool for human endometrial receptivity [42]. *TBC1D8* is expressed in the embryo and endometrium of Holstein cattle [43]. *CRACR2B* is related to sire stillbirths. It is a regulator of the calcium release activation channel and belongs to the calcium-ion binding signaling pathway. Abortion in dairy cows is closely related to the calcium signaling pathways. Calcium ions are important messengers involved in the normal development and function of the placenta [44].

As the first detailed analysis of *cis*-e/sQTL during early to mid-lactation in dairy cattle, our study has certain limitations. The number of samples and the density of SNPs need to be increased to improve the detection power and number of e/sQTLs. Meanwhile, the identification of molecular QTLs in bulk tissue will mask the effects of some QTLs, and analyzing molecular QTLs at the single-cell level will help us better understand the impact of regulatory variation on complex traits.

## Conclusions

This study demonstrated the importance of considering the lactation stage of blood expression when using eQTL and sQTL data to interpret complex trait-associated variants in dairy cattle. Blood samples can help us understand the regulatory mechanism of eQTL and sQTL on the complex traits of dairy cattle in early to mid-lactation, and the identified important SNPs and genes can provide reference for downstream molecular experimental verification and application.

## Materials and methods

### Sample collection and phenotyping

A total of 104 blood samples of Holstein cows in early to mid-lactation (22–150 days after calving and parity ≤ 3) were collected from the tail vein and stored in EDTA vacutainers for blood routine testing, genotyping and RNA-Seq. Milk was collected three times daily at a ratio of 4:3:3, with each collection consisting of a mixed sample from the four milk quarters of the cow. This was then stored in a tube with preservatives at 4℃ for determination of milk quality. All procedures involving experimental animals were approved by the Animal Welfare Committee of the China Agricultural University, Beijing, China. All efforts were made to minimize suffering and discomfort of the experimental animals.

### RNA extraction, sequencing, and quality control

The white blood cell layer was separated from fresh anticoagulated blood and centrifuged at 3,500 rpm for 15 min. Total RNA was isolated from peripheral blood leukocytes using TRIzol (Thermo Fisher Scientific, Waltham, MA, USA) according to the manufacturer’s instructions. RNA quality was checked using a 1% agarose gel and a NanoDrop. The integrity of RNA (RIN) was tested using an Agilent 2100, and quantification was performed using a Qubit 2.0. Sequencing was completed on an Illumina NovaSeq-6000 platform, and 150-bp paired-end reads were produced. Trimmomatic (v0.39) [45] was used to perform quality control of raw reads using the following parameters: adapters: TruSeq3-PE.fa:2:30:10 LEADING:3 TRAILING:3 SLIDINGWINDOW:4:15 MINLEN:36.

### DNA genotyping and quality control

Individuals were genotyped using a GGP bovine 150 K BeadChip (Neogen, Lansing, MI, USA). SNPs with a minor allele frequency < 0.05, Hardy-Weinberg equilibrium exact test *P*-value less than 1 × 10^− 5^, missing genotype rates per-variant > 0.05 and missing genotype rates per-sample > 0.1 were filtered from analysis. The SNPs coordinates of the 150 K BeadChip were transferred from UMD3.1 to ARS.UCD.1.2, based on the SNP rsid. Ultimately, 101 individuals and 95,799 SNPs were included in the analysis.

### Quantification of gene expression and alternative splicing

Qualified RNA-seq reads were aligned to the ARS.UCD.1.2 genome [46] using STAR (v2.7.9a) [47]. Mapped reads were used for quantification and normalization of gene expression. TPM (Transcripts per million) were obtained using StringTie (v2.1.5) [48] and transcript counts were quantified using featureCounts (subread package v2.0.2) [49]. To improve the reliability of our data, we only saved genes with TPM > 0.1 in at least 20% of the samples, and read counts greater than 6 in at least 20% of the samples were retained. EdgeR (v3.34.1) [50] was used to perform Trimmed Mean of M-values (TMM) and Counts per million (CPM) normalization on these genes. Inverse normal transformed values of gene expression were obtained for downstream analysis.

LeafCutter (v.0.2.9) [51] was used to identify and quantify the variable alternative splicing events of the genes. First, the bam files obtained from STAR alignment is converted into junction files using the script ‘bam2junc.sh’. Then, the script ‘leafcutter_cluster.py’ was used to performed intron clustering with default settings of 50 reads per cluster and a maximum intron length of 500 kb. Afterward, we conducted the ‘prepare_genotype_table.py’ script in LeafCutter to calculate intron excision ratios and to remove introns used in fewer than 40% of individuals or with no variation. Finally, the standardized and quantile-normalized intron excision ratios were used as the percent spliced-in (PSI) values across samples.

### Covariate analysis for QTL discovery

To remove the effects of hidden batch effects and other biological sources of transcriptome-wide variation in gene expression and intron excision ratios, we applied the PEER method (probabilistic estimation of expression residuals) [20] to identify and account for additional covariates based on the matrix of gene expression and intron excision ratio, respectively. The top five genotype principal components (PCs) were calculated using SNPRelate (v1.26.0) [52] to account for the effects of population genetic structure. Pearson correlations between the top ten gene expression PCs (EPC), intron excision ratio PCs (SPC), and known phenotypes (week of lactation, parity, RIN and blood cell count) were calculated to identify other factors that affect the expression level of the molecular phenotype. Week of lactation, parity, and the RNA integrity number (RIN) showed the highest correlations and were used as known covariates. Finally, the top five genotype PCs, week of lactation, parity, RIN, and PEER factors (the top 10 peer factors in *cis*-eQTL mapping and the top eight peer factors in *cis*-sQTL mapping) were removed from the QTL mapping.

### Estimation of the heritability of gene expression and intron excision ratio

A total of 14,303 genes and 227,733 intron clusters were used to estimate the heritability of gene expression and the intron excision ratio, respectively. The *cis*-SNPs used to estimate the heritability of gene expression were defined as SNPs within 1 Mb of the target gene transcription initiation site (TSS), while *cis*-SNPs used to estimate the heritability of the intron excision ratio were defined as SNPs within 1 Mb of the target intron clusters. GCTA (v1.93.3 beta2) [53] was used to generate the corresponding genetic relationship matrix (GRM) based on the *cis*-SNPs of target genes or intron clusters. Subsequently, heritability was estimated by using the restricted maximum likelihood (REML) algorithm through the “-reml” function in GCTA while correcting for the aforementioned covariates.

### *cis*-eQTL mapping

We used a linear regression model in FastQTL (v2.184) [54] to test the associations of the expression levels of genes with SNPs within TSS 1 Mb of target genes, while adjusting for the corresponding top 10 PEER factors, top five genotype PCs, and the known covariates (week of lactation, parity and RIN). This method is consistent with cGTEx [4]. First, *cis*-eQTL mapping was performed in permutation mode to identify genes (eGene) with at least one significant *cis*-eQTL. The *cis*-eQTLs *FDR* ≤ 0.05 were considered as significant, calculated using the Benjamini–Hochberg method based on the beta distribution-extrapolated empirical *P-*values from FastQTL. To identify a list of significant eQTL-eGene pairs, the nominal mode was applied to FastQTL. The genome-wide empirical *P*-value threshold *p*_*t*_ for each gene was defined as the empirical *P*-value of the gene closest to an 0.05 *FDR* threshold. We then calculated the nominal threshold as *F*^− 1^(*p*_*t*_) for each gene using the permutation mode of FastQTL (v2.184), where *F*^− 1^ is the binominal inverse cumulative distribution. Variants with nominal *P*-values below the nominal threshold as significant and included in the list of eGene–eVariant pairs.

### *cis*-sQTL mapping

The *cis*-sQTL mapping was performed with FastQTL, testing for associations with SNP within ± 1 Mb of target intron clusters and their corresponding intron excision ratio. The covariates used were the same as *cis*-eQTL mapping, except that the top eight PEER factors were used in *cis*-sQTL mapping. Unlike *cis*-eQTL mapping, grouped permutations were used to jointly compute empirical *P*-value for all intron clusters. The top nominal *cis*-sQTL for a gene was defined as the highest association among all assigned clusters and introns. The 1,000–10,000 permutations were applied in FastQTL to obtain beta-approximated permutation *P*-values. The sQTL–intron pairs *FDR* ≤ 0.05 were considered as significant, and defined *cis*-sGene as genes containing any introns with a significant *cis*-sQTL. To identify *cis*-sGenes, similar to *cis*-eQTLs, computation of an sGene-level nominal *P*-value threshold was used to identify all significant variant-intron pairs.

### Enrichment analysis of eQTL and sQTL

Ensembl Variant Effect Predictor (VEP) was used to annotate the effects of variants. Additionally, annotations of genomic chromatin states in cow spleen were used for enrichment analysis [55]. Compared to all SNP loci on the beadchip, Fisher’s exact test is conducted on eQTLs and sQTLs to determine whether they are significantly enriched at these loci or regions.

### Comparison of blood *cis*-eQTL between Holstein in early-mid lactation and cGTEx cattle

To determine the specific *cis*-eQTLs and eGenes in the early to mid-lactation period of Holsteins, the SNPs detected exclusively in this study and not in cGTEx were firstly removed. Subsequently, the *cis*-eQTLs (*P* < 0.05) and eGenes identified in this study were compared with the *cis*-eQTLs (*P* < 0.05) and eGenes of cGTEx [4] for SNP overlap analysis.

### WGCNA (weighted gene co-expression network analysis) and enrichment analysis of eGenes

Co-expression modules of 3,419 eGenes (*P* < 0.05; this study) were built with a soft threshold of 5 to explore the relationship between underlying modules and some phenotypes, including six blood counts from routine blood tests (leukocyte count, neutrophil ratio, lymphocyte ratio, monocyte ratio, eosinophil ratio, and basophil ratio) and milk composition records. The eGenes of modules associated with at least one phenotype were used for GO (Gene Ontology) enrichment analysis using the online website KOBAS-i [56].

### Colocalization analysis and transcriptome-wide association study (TWAS)

To test whether eQTL and sQTL co-localized with GWAS signals of complex traits in dairy cows, we used the GWAS summary statistics of milk yield, milk fat yield, milk protein yield, and somatic cell score from 27,214 bulls for colocalization analysis [21]. The Bayesian based software Coloc (v5.1.0) [57] was used for the analysis. PP4 (posterior probability of colocalization hypothesis) > 0.60 in eQTL and sQTL, was used to determine colocalization.

TWAS was performed to estimate the association between gene expression levels and complex traits using S-PrediXcan (v0.6.11) [58]. In this study a nested cross-validated Elastic Net prediction model was first trained based on the genotype and normalized gene expression data of 101 individuals in this study. In addition to the GWAS summary statistics used for colocalization [21], retained placenta, productive life, metritis, mastitis, livability, ketosis, hypocalcemia, sire calving ease, and sire stillbirth were used for TWAS [59]. Genes with *P* < 0.05 were considered to be significantly correlated with these traits.

### Electronic supplementary material

Below is the link to the electronic supplementary material.

Supplementary Material 1

Supplementary Material 2

## Data Availability

The raw RNA sequence data reported in this paper have been deposited in the Genome Sequence Archive in National Genomics Data Center, China National Center for Bioinformation / Beijing Institute of Genomics, Chinese Academy of Sciences (GSA: CRA012735) that are publicly accessible at https://ngdc.cncb.ac.cn/gsa/s/saqR97XE. The public GWAS summary statistics from Figshare (https://figshare.com/s/ea726fa95a5bac158ac1). The public eQTL summary statistics from cGTEx (https://cgtex.roslin.ed.ac.uk/). The SNP BeadChip data for the current study are available from the corresponding author on reasonable request.
