# Supplementary material for: Identification and characterization of whole blood gene expression and splicing quantitative trait loci during early to mid-lactation of dairy cattle
Source: BMC Genomics. 2024 May 6;25:445. doi: 10.1186/s12864-024-10346-7 (PMC11075310; doi:10.1186/s12864-024-10346-7)
Supplement: Supplementary file 1 — Supplementary Material 1 [file 12864_2024_10346_MOESM1_ESM.docx]

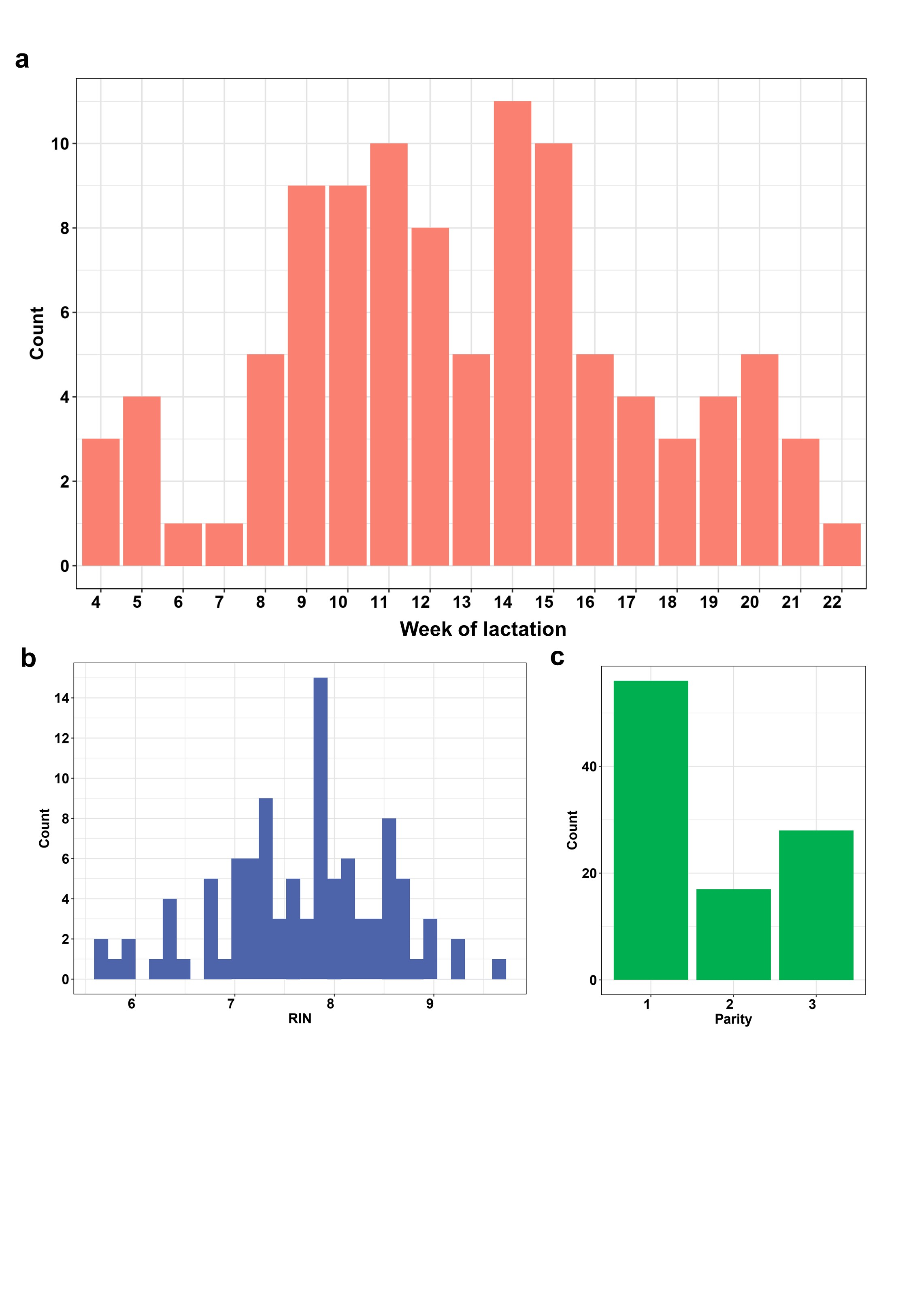


Figure S1. Week of lactation (a) RIN (b) and parity (c) of 101 Chinese Holstein cattle during early to mid-lactation.


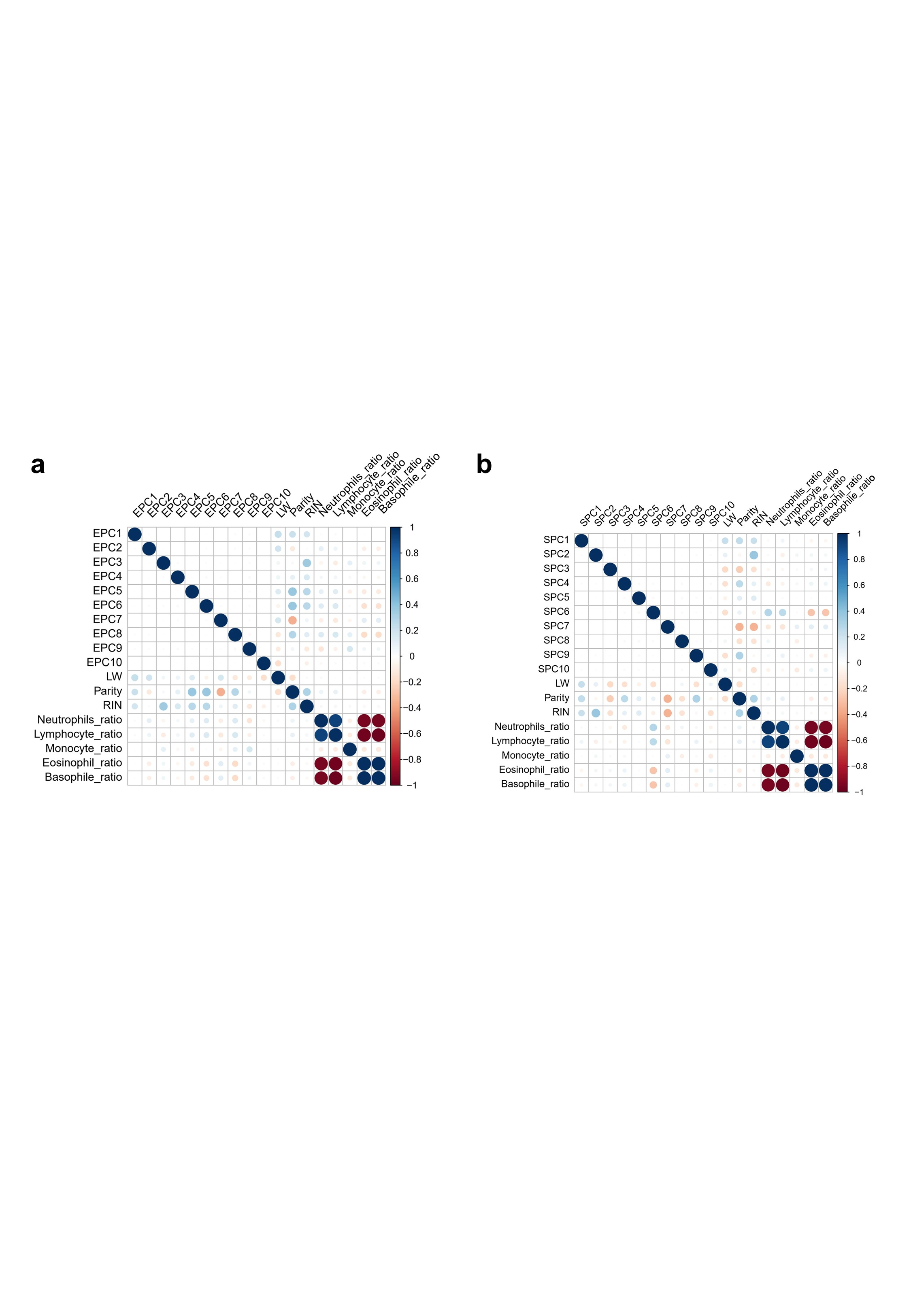


Figure S2. (a) Correlation between EPCs and individual phenotypes. (b) Correlation between SPCs and individual phenotypes. LW: week of lactation


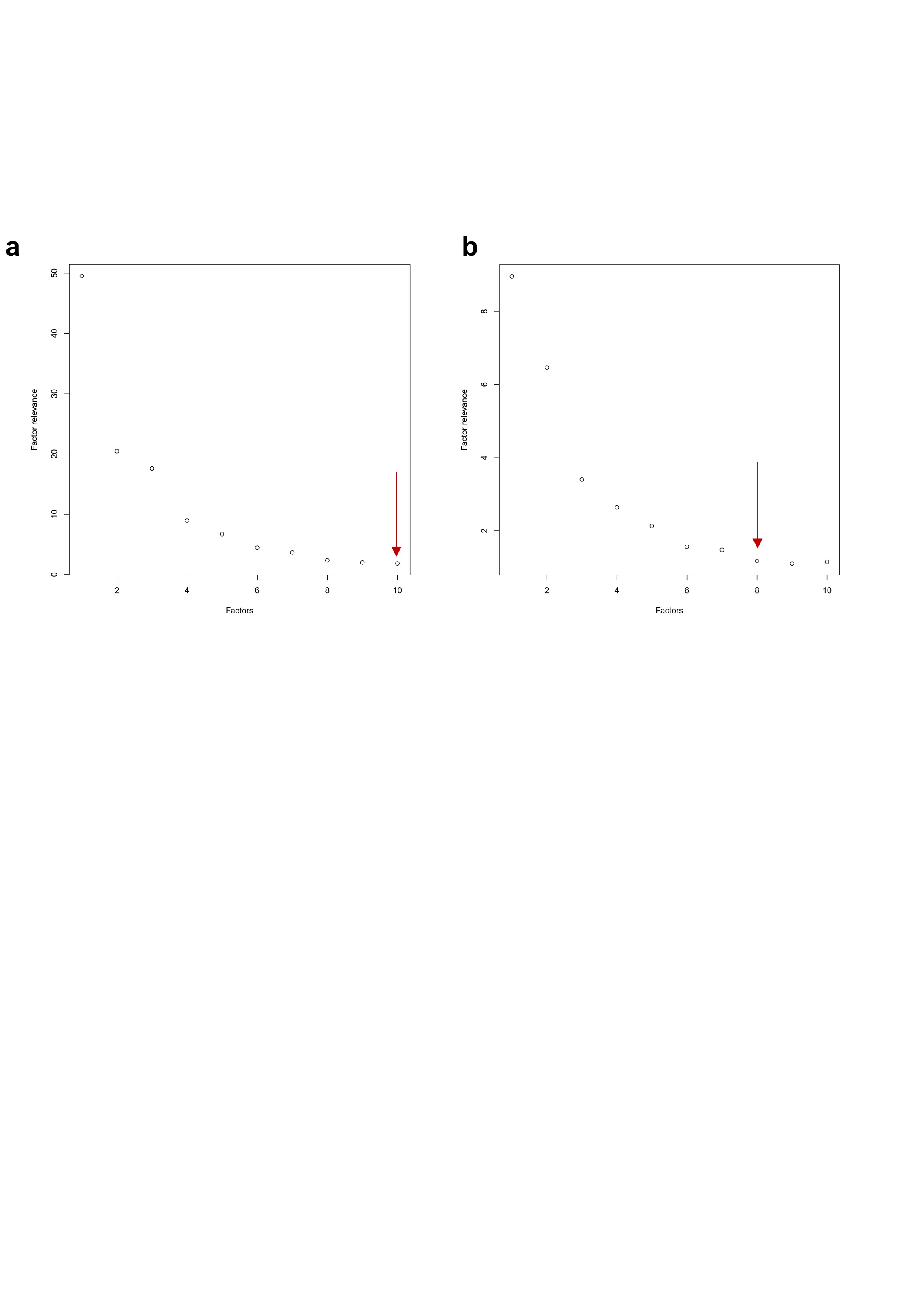


Figure S3. PEER factor relevance for eQTL (a) and sQTL (b) identification.


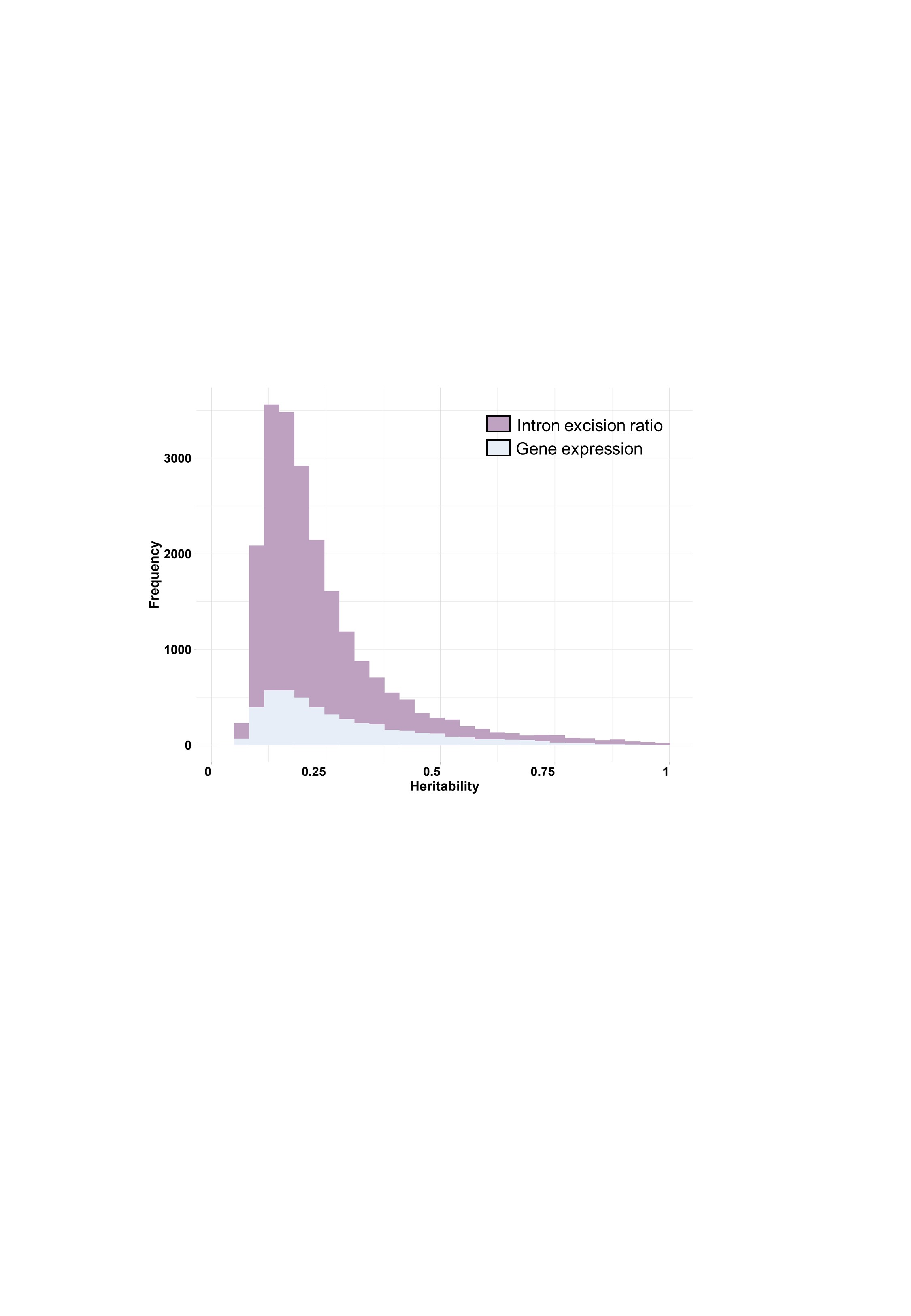


Figure S4. Heritability of 4,604 genes expression and 21,983 intron excision ratio explained by *cis*-SNPs.


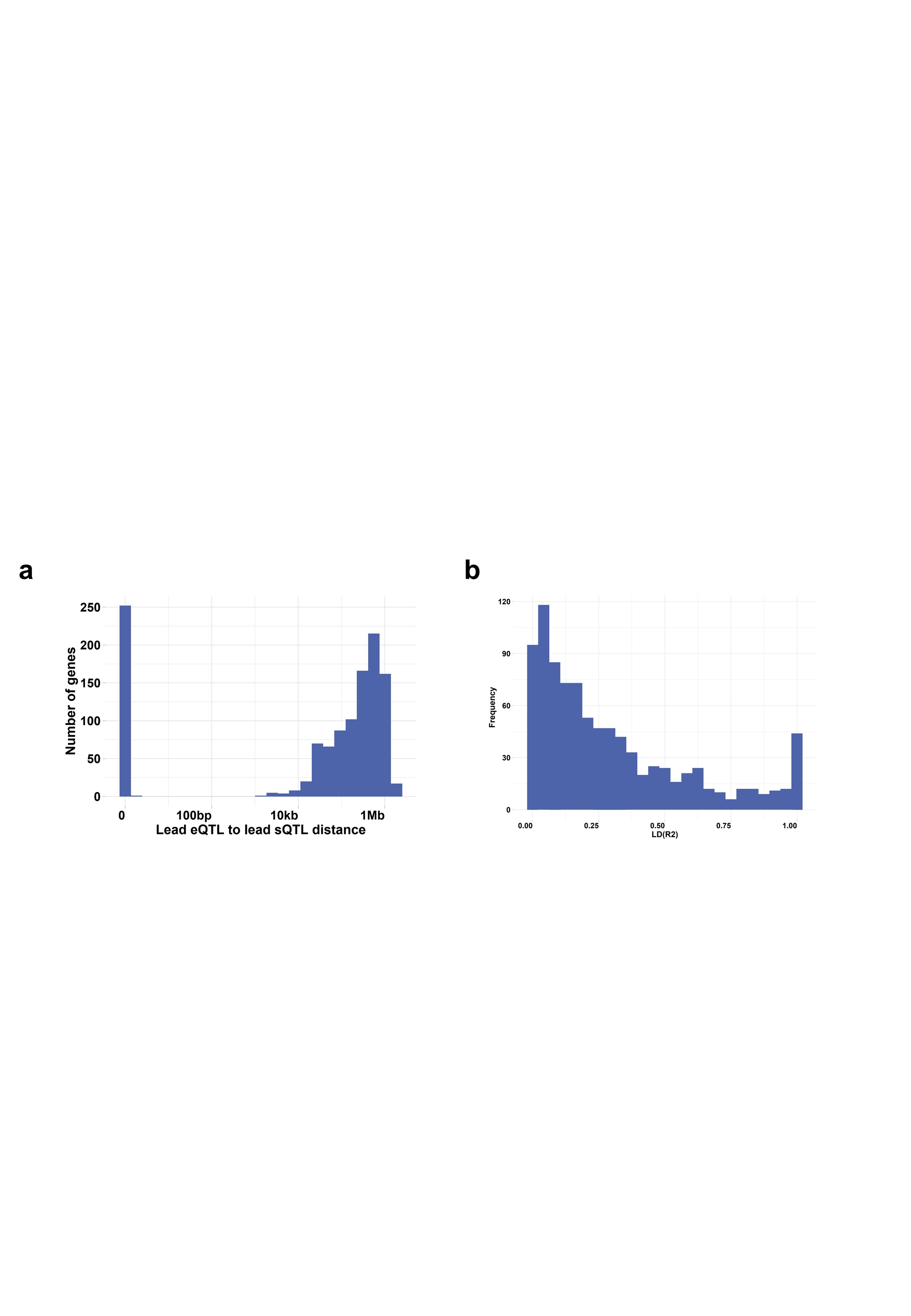


Figure S5. (a) The physical distance between the lead eQTL and lead sQTL corresponding to genes that are both eGene and sGene. The significance level of all cis-eQTL and cis-sQTL in this figure is FDR < 0.05. (b) The LD r^2^ between the lead eQTL and lead sQTL corresponding to genes that are both eGene and sGene.


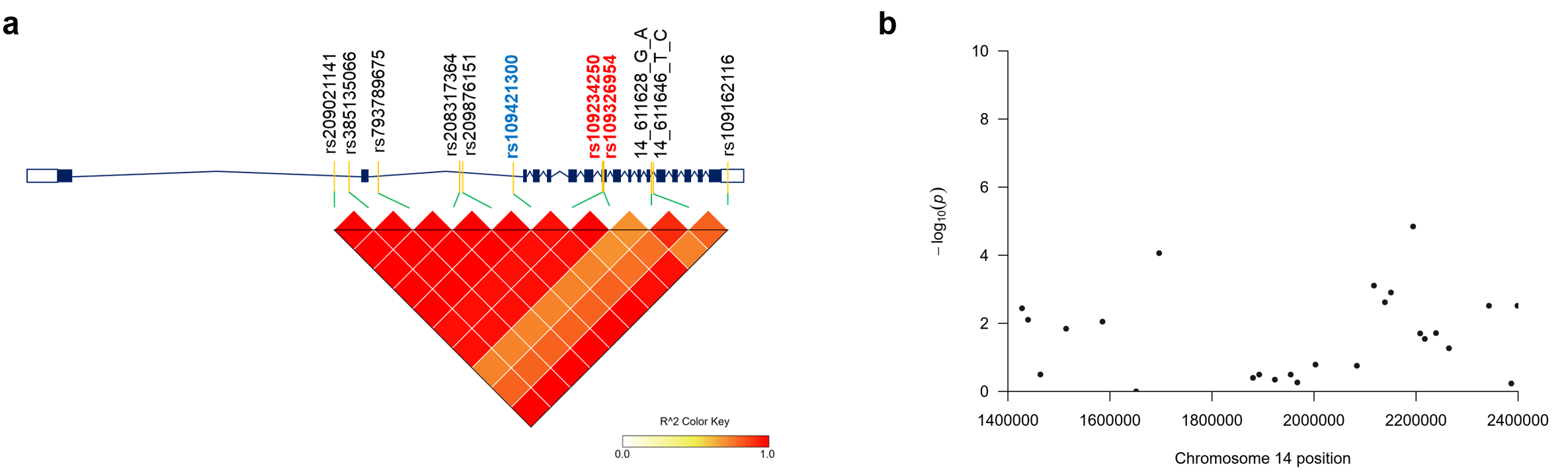


Figure S6. (a) Linkage disequilibrium analysis of SNPs in *DGAT1* gene region. The SNP data is derived from the whole-genome resequencing (coverage > 10×) of 105 Holstein cows. Red represents the K232A coding mutation, and blue represents the variant obtained from the colocalization analysis in this study. (b) SNP rs109421300 was used as a covariate for conditional analysis of *DGAT1* gene expression.
